# Membrane-bound ICAM-1 contributes to the onset of proinvasive tumor stroma by controlling acto-myosin contractility in carcinoma-associated fibroblasts

**DOI:** 10.18632/oncotarget.13610

**Published:** 2016-11-25

**Authors:** Stephanie Bonan, Jean Albrengues, Eloise Grasset, Sanya-Eduarda Kuzet, Nicolas Nottet, Isabelle Bourget, Thomas Bertero, Bernard Mari, Guerrino Meneguzzi, Cedric Gaggioli

**Affiliations:** ^1^ INSERM U1081, CNRS UMR7284, Institute for Research on Cancer and Aging, Nice (IRCAN), University of Nice Sophia Antipolis, Medical School, F-06107, Nice, France; ^2^ Institut de Pharmacologie Moléculaire et Cellulaire (IPMC), CNRS UMR7275, Sophia-Antipolis, France

**Keywords:** carcinoma-associated fibroblast, ICAM-1, tumor microenvironment, inflammation, extracellular matrix

## Abstract

Acto-myosin contractility in carcinoma-associated fibroblasts leads to assembly of the tumor extracellular matrix. The pro-inflammatory cytokine LIF governs fibroblast activation in cancer by regulating the myosin light chain 2 activity. So far, however, how LIF mediates cytoskeleton contractility remains unknown. Using phenotypic screening assays based on knock-down of LIF-dependent genes in fibroblasts, we identified the glycoprotein ICAM-1 as a crucial regulator of stroma fibroblast proinvasive matrix remodeling. We demonstrate that the membrane-bound ICAM-1 isoform is necessary and sufficient to promote inflammation-dependent extracellular matrix contraction, which favors cancer cell invasion. Indeed, ICAM-1 mediates generation of acto-myosin contractility downstream of the Src kinases in stromal fibroblasts. Moreover, acto-myosin contractility regulates ICAM-1 expression by establishing a positive feedback signaling. Thus, targeting stromal ICAM-1 might constitute a possible therapeutic mean to counteract tumor cell invasion and dissemination.

## INTRODUCTION

Carcinoma-associated fibroblasts (CAF) are the most representative non-cancerous cell population of the tumor stroma. In several instances, presence of CAF dictates the tumor outcome [1–4]. CAF participate to all steps of carcinomagenesis, from tumor initiation to metastatic spreading in secondary organs, essentially by generating a proinvasive tumoral stroma that favors tumor cell propagation and dissemination from the primary tumor [5–8]. Indeed, secretion of inflammatory molecules, including chemokines of the IL6 family, triggers a proinvasive fibroblast activation. In such context, we have demonstrated the crucial role that Leukemia Inhibitory Factor (LIF) plays in the proinvasive ECM remodeling by inducing acto-myosin contractility in fibroblasts [9]. In fibroblasts, LIF activates the GP130/JAK1/STAT3 signaling pathway, which initiates tensile force generation through regulation of the RhoA/ROCK/MLC2 signaling pathway. Moreover, in activated fibroblasts and CAF, constitutive activation and crosstalk of these two signaling pathways lead to the generation of fibrotic and tumorigenic cancer-associated ECM [10]. However, the LIF-dependent genes that mediate the crosstalk between inflammation and acto-myosin contractility in fibroblast remain to be identified.

Intercellular Adhesion Molecule 1 (ICAM-1), a member of the immunoglobulin superfamily, is a cell surface glycoprotein receptor for LFA-1 (Lymphocyte Function-associated Antigen 1) and MAC-1 (Macrophages Adhesion Ligand 1) integrins, but also for ECM proteins [11–16]. ICAM-1 is considered to be an inflammatory responsive gene, whose expression is highly induced in injured tissues [17, 18]. The membrane-bound ICAM-1 isoform is expressed at the cell surface of a variety of cell types including endothelial, epithelial, immune cells and fibroblasts [19–21], but it can also be found as a soluble secreted form (sICAM-1) [22–24]. ICAM-1 is mainly responsible for intercellular adhesion and trafficking of inflammatory cells. Membrane-bound ICAM-1 engagement at the cell surface results in an out-side-in signaling triggered by the activation of specific tyrosine kinases, which, depending on the cellular contexts, leads to transcription factors activation, inflammation, production of reactive oxygen species and cell proliferation [19]. Membrane-bound ICAM-1 expression by colorectal cancer cells has been associated with reduced tumor cell dissemination and metastatic potential [25, 26]; whereas ICAM-1 expression by stromal fibroblasts suggests a tumor promoting effect potentially through increased monocytic cell recruitment to the tumor mass [27]. Yet, the mechanisms by which CAF-associated ICAM-1 acts as a tumor promoter remain unclear.

Using organotypic cell cultures submitted to three-dimensional collagen-rich contraction assays after RNAi-mediated knock down of LIF-responsive genes, we show that membrane-bound ICAM-1 triggers tumorigenic ECM remodeling in CAF and in fibroblasts undergoing activation by tumor cells. ICAM-1 is thus identified as a crucial regulator of the inflammation-dependent ECM remodeling in cancer. We demonstrate that ICAM-1 promotes proinvasive ECM remodeling through regulation of acto-myosin contractility. Moreover, inhibition of acto-myosin contractility in CAF induces a decrease of ICAM-1 expression, which suggests that a positive feedback signaling mediates excessive ECM deposition and fibrotic tissue formation in cancers. We also show that CAF are heterogeneous for the membrane-bound ICAM-1 expression at the cell surface, and that such expression is specific to sub-sets of contractile and proinvasive CAF populations. Finally, we demonstrate that ICAM-1 is expressed in the tumor stroma of human head and neck cancers in correlation with the presence of clusters of invasive cancer cells.

## RESULTS

### Long-term TGFβ-activated fibroblasts display a LIF-dependent gene signature

**Figure 1:**
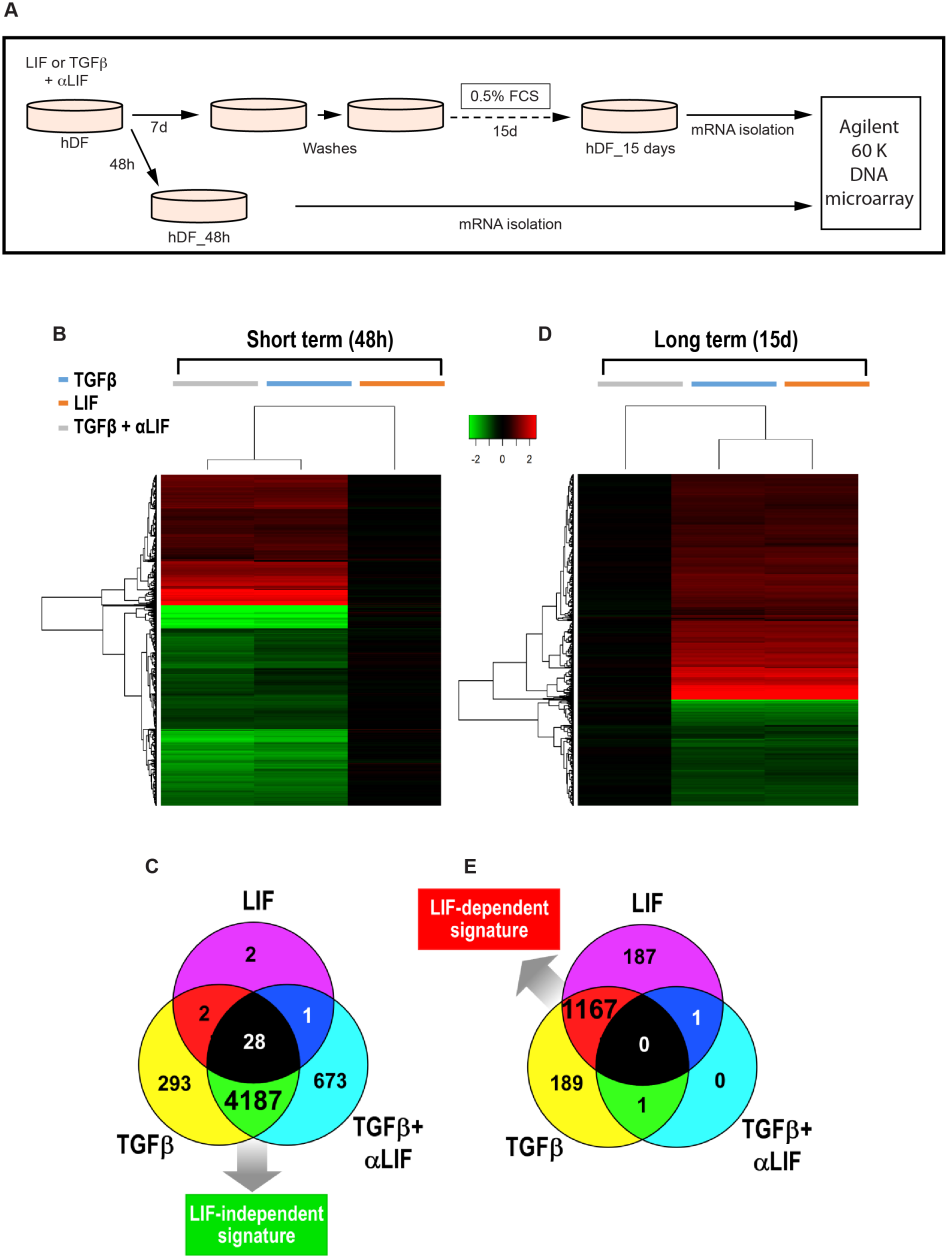
LIF supports long-term TGFβ-activated fibroblasts transcriptomic signature **A.** Schematic representation of the experimental design of the short- or long-term hDF stimulation by LIF or TGFβ in presence or absence of LIF blocking antibody (αLIF). Short-term cytokines stimulation: **B.** Heatmaps comparing the normalized log2 ratio between stimulated hDF versus control cells at short-term. **C.** Venn diagrams showing the overlapping set of genes regulated (both up-regulated and down-regulated) by the three experimental conditions at short-term. **D.** Heatmaps comparing the normalized log2 ratio between stimulated hDF versus control cells at long-term. **E.** Venn diagrams showing the overlapping set of genes regulated (both up-regulated and down-regulated) by the three experimental conditions at long-term.

To unveil the genes controlling acto-myosin cytoskeleton contractility in stromal fibroblasts and thus involved in generation of a proinvasive matrix, we first conducted a pan-genomic transcriptome analysis of human primary dermal fibroblasts (hDF) following a short- (48 hours) or long-term (15 days) *in vitro* stimulation by TGFβ or LIF (Figure 1A). A short-term stimulation led to distinct transcriptomic changes with only 33 genes significantly modulated by LIF, while TGFβ induced a wide transcriptomic response involving several thousand genes (Figure 1B and 1C). In fibroblasts stimulated by TGFβ, a LIF blocking antibody (αLIF) failed to alter response to TGFβ (Figure 1B and 1C). Conversely, in the case of the long-term stimulation, LIF or TGFβ induced a comparable signature (Figure 1D) with more than 1,000 genes significantly regulated by both factors (Figure 1E). However, addition of the LIF-blocking antibody completely inhibited the TGFβ effect (Figures 1B, right panel and Figure 1C), which unveiled a transcriptomic switch from an early TGFβ-specific to a long-term LIF-dependent gene signature. The pivotal role of LIF in the gene modulation associated with maintenance of the proinvasive phenotype acquired by the long-term TGFβ-activated fibroblasts was thus demonstrated. We next assessed whether such a LIF-dependent gene signature is also shared by the CAF isolated from tissue biopsies of patients with head and neck, lung or breast carcinomas. A subset of 10 genes (eight up-regulated and two down-regulated) was selected, firstly to confirm the microarray data by qRT-PCR analysis in both TGFβ and LIF activated fibroblasts (Supplementary Figure S1A) and secondly for comparative mRNA quantification analysis with CAF and the hDF control (Supplementary Figure S1B). qRT-PCR analysis confirmed that both LIF and TGFβ regulate expression of all the 10 genes that, importantly, are similarly regulated in CAF. These findings demonstrate that the genes regulated by LIF in *in vitro* TGFβ-activated fibroblasts are similarly regulated in CAF.

### Membrane-bound ICAM-1 governs the onset of proinvasive ECM

**Figure 2:**
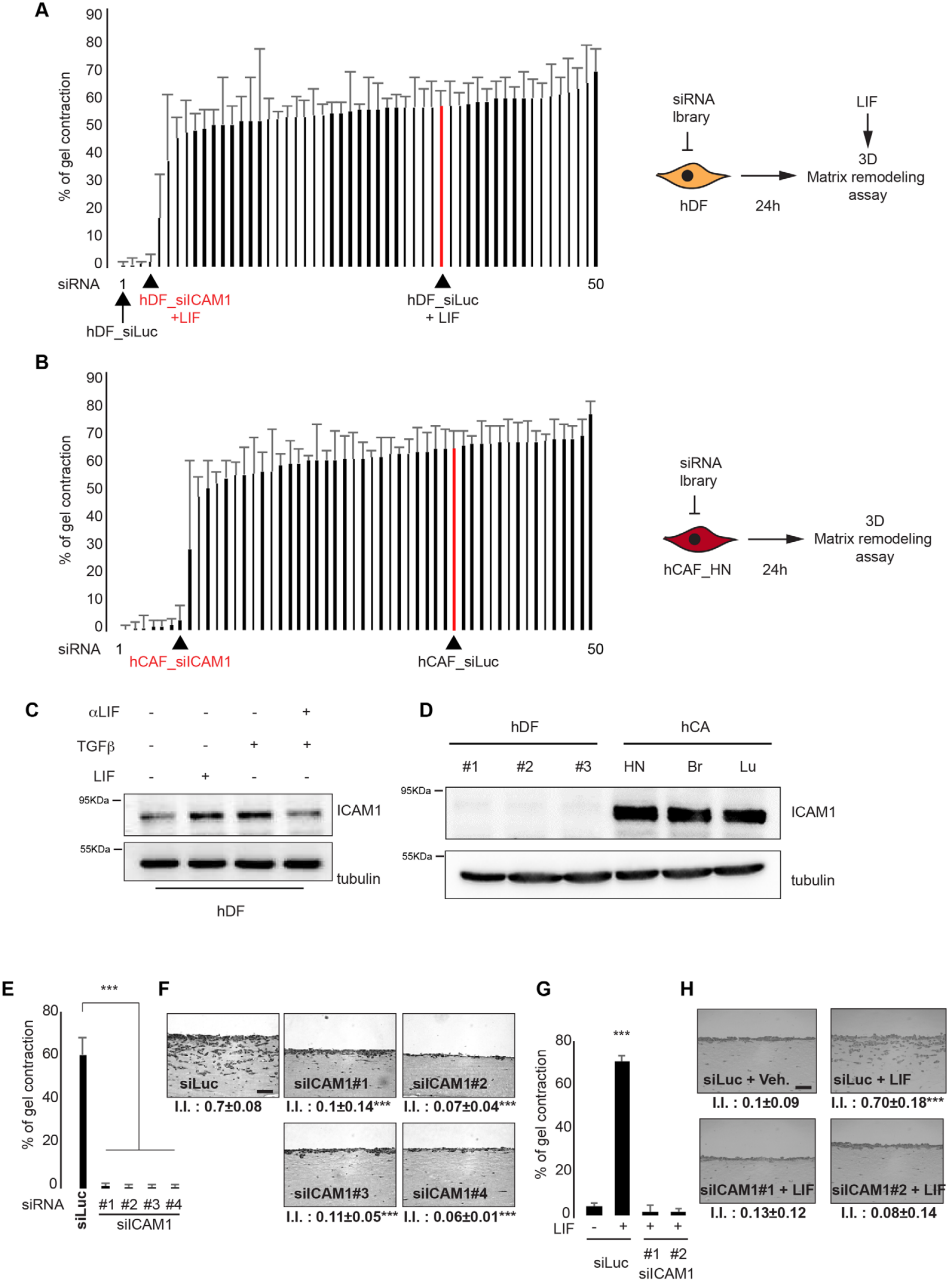
ICAM-1 controls proinvasive ECM remodeling **A.** Percentage of gel contraction by hDF six days after RNAi transfection and subsequent LIF stimulation (left panel, n=2 in triplicates). Schematic representation of the experimental conditions (right panel). **B.** Percentage of gel contraction by CAF 6 days after RNAi transfection (left panel, n=2 in triplicates). Schematic representation of the experimental conditions (right panel). **C.** Immunoblot of ICAM-1 in hDF following LIF or TGFβ1 stimulation, in the presence (48h) or absence of the LIF blocking antibody. Immunoblot of tubulin was the internal control. **D.** Immunoblot of ICAM-1 in three hDF #1, #2 and #3) and in three CAF (Head and Neck, Breast and Lung). Immunoblot as control. **E.** Percentage of gel contraction by CAF transfected with control RNAi (siLuc) or RNAi targeting ICAM-1 (siICAM-1#1, #2, #3 and #4) (n=3 in triplicates, mean + s.d, ***p<0.001). **F.** Representative images of H&E coloration of paraffin-embedded sections of SCC12 3D-cultures in response to CAF transfected with control (siLuc) or targeting ICAM-1 (siICAM-1#1, #2, #3 and #4) siRNA (n=3, I.I., invasion index, mean ± s.d., ***P<0.001). Scale bar 100μm. **G.** Percentage of gel contraction by LIF-stimulated or not (Veh.) HDF subsequently transfected with control (siLuc) or ICAM-1-targeting ICAM-1 (siICAM-1#1 and #2) RNAi (n=3 in triplicates, mean + s.d, ***p<0.001). **H.** Representative images of H&E staining of paraffin-embedded sections of SCC12 in response to control (veh) or LIF-activated hDF subsequently transfected with control (siLuc) or ICAM-1-targeting siICAM-1#1 and #2) RNAi ((n=3, I.I., invasion index, mean ± s.d., ***P<0.001). Scale bar 100μm.

Having demonstrated that LIF induces and sustains a contractile and proinvasive phenotype in activated fibroblasts [9, 28], we speculated that the genes essential for fibroblast acto-myosin cytoskeleton contractility, and thus for CAF-dependent proinvasive matrix remodeling, could be transcriptionally regulated by LIF. Accordingly, the LIF-blocking antibody is expected to inhibit contractility of *in vitro* long-term TGFβ-stimulated fibroblasts. A three-dimensional RNAi-based phenotypic screening was thus set up to identify the genes governing onset of CAF-dependent proinvasive ECM remodeling that strongly correlates with matrix contraction [29]. In light of our results on the LIF-dependent up-regulation of fibroblasts and the inhibitory effect of the LIF-blocking antibody on TGFβ-stimulated hDF, 50 genes were selected on the basis of their known or putative biological functions described in the literature on ECM remodeling, cytoskeleton organization, cell contractility, metabolism and transcription (Figure 1E in red and Supplementary Table S1A). Using hDF and CAF, three independent screenings were implemented to identify genes that specifically regulate the initiation and the maintenance phase of the cell contractility activation by LIF (Figures 2A-2C and Supplementary Table S1B-S1E). Thus, hDF were transfected with smart pools of RNAi targeting the 50 selected genes (Supplementary Table S2). Non-targeting RNAi were negative controls, while RNAi targeting the JAK1 kinase were positive controls. The next day, hDF were embedded in a three-dimension collagen lattices, then low serum media supplemented with LIF was added. Six days later, gel contraction was quantified, which revealed four genes (HRH1, DBC1, BCL3 and ICAM-1) essential for initiation of LIF-dependent contractility in fibroblasts (Figure 2A, Supplementary Tables S1B and S1E). Next, the genes sustaining the contractile activity in long-term LIF-activated hDF were investigated. HDF were activated *in vitro* for 15-days, then transfected using the RNAi smart pools. Collagen lattice contraction and quantification were then assessed as above. Six genes (HRH1, DBC1, BCL3, ICAM-1, GGT5 and ANGPTL4) appeared to be crucial for the maintenance of the LIF-dependent contractility of hDF (Supplementary Figure S2A, Supplementary Tables S1C and S1E). To confirm the LIF-dependent gene signature and, more specifically, the role of the identified genes in CAF contractility, CAF isolated from human head and neck carcinoma were transfected using the 50 RNAi bank and embedded in collagen lattices 24 hours later. Six genes (HRH1, DBC1, BCL3, ICAM-1, ANGPTL4 and BCL2L14) resulted to be crucial for CAF-dependent collagen lattice contraction (Figure 2B, Supplementary Tables S1D and S1E). Interestingly, most of the genes found to be crucial in LIF-activated hDF also support CAF contractility. RNAi to genes HRH1, DBC1, BCL3, ICAM-1 consistently blocked the activated fibroblast matrix contraction with no significant impact on CAF viability (data not shown).

Because the membrane-bound adhesion molecule ICAM-1 may serve as a preferential target for immune-cancer therapies, its potential role in the CAF-dependent onset of a proinvasive ECM remodeling was further analyzed. We first confirmed that ICAM-1 is induced by both LIF and TGFβ (Figure 2C) and showed that ICAM-1 expression in hDF is stimulated by tumor cells conditioned media (Supplementary Figure S2B). Interestingly, ICAM-1 appeared overexpressed in CAF isolated from head and neck, lung and breast carcinomas when compared to hDF (Figure 2D). The role of membrane-bound ICAM-1 in CAF-dependent SCC cell collective invasion was then assessed using organotypic three-dimensional invasion assays. Inhibition of ICAM-1 expression in CAF by specific knock-down expression by four independent RNAi oligonucleotides confirmed the involvement of ICAM-1 in matrix contraction (Figure 2E and Supplementary Figure S2C) and also revealed the crucial role for ICAM-1 in the onset of a proinvasive ECM remodeling (Figure 2F and Supplementary Figure S2C). Additionally, ICAM-1 was found to support LIF-dependent contractile and proinvasive fibroblast activation (Figures 2G-2H and Supplementary Figure S2D). Interestingly, interfering in both CAF and LIF-activated fibroblasts using a specific anti-ICAM-1 blocking antibody (αICAM-1) dramatically reduced both collagen gel contraction and collective invasion of SCC12 cells (Supplementary Figures S2E-S2G). Taken together, these data identify membrane-bound ICAM-1 as a crucial regulator of contractile and proinvasive CAF activities, and highlight a novel potential therapeutic target for the procarcinogenic activity of CAF in cancer development.

### Membrane-bound ICAM-1 triggers inflammation-dependent cancerous ECM

**Figure 3:**
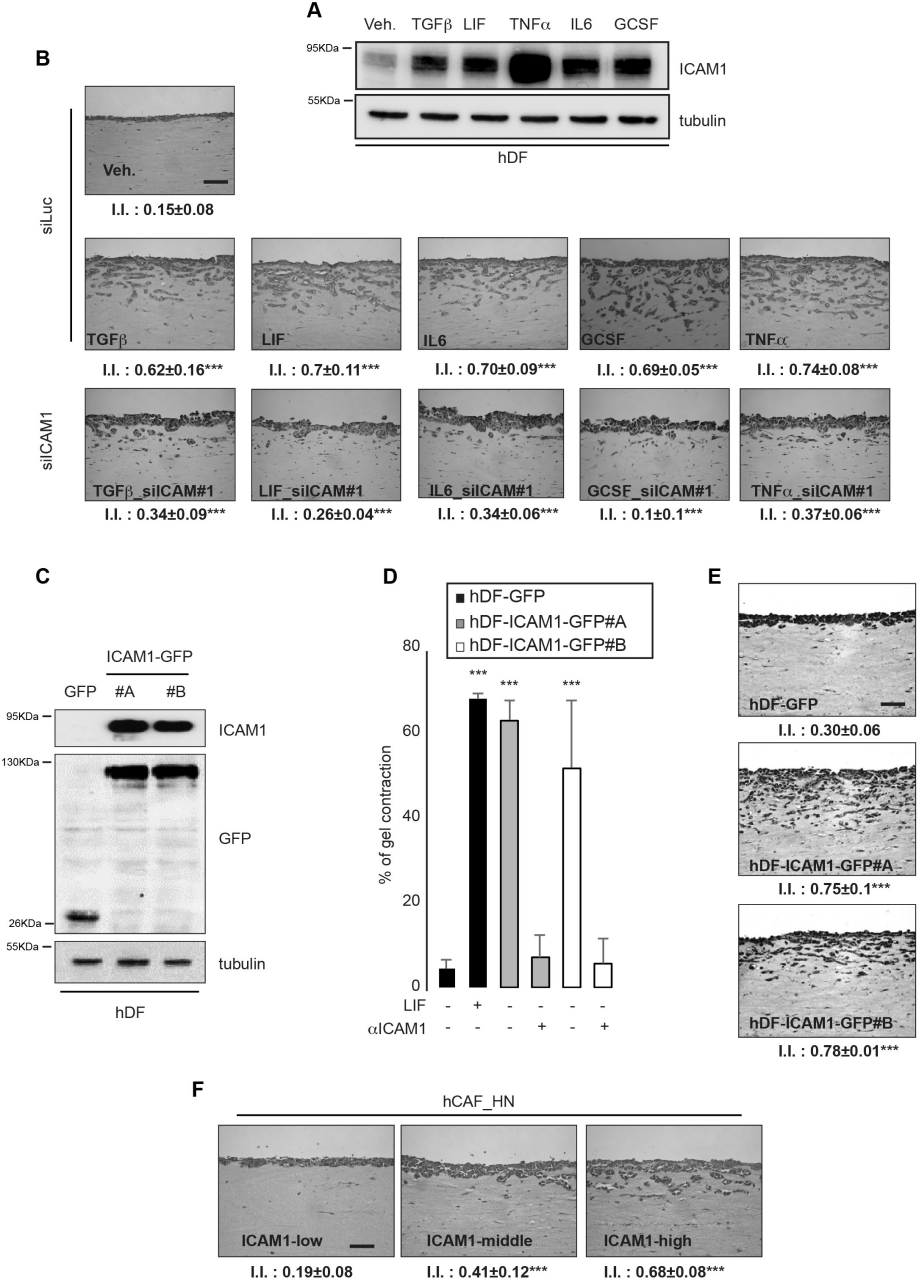
ICAM-1 drives inflammation-dependent proinvasive ECM remodeling **A.** Immunoblot of ICAM-1 in hDF following cytokines stimulation for 24 hours. Immunoblot of tubulin shown as control. **B.** Representative images of H&E staining of paraffin-embedded sections of SCC12 in response to control hDF (Veh.; siLuc) or HDF stimulated by inflammatory cytokines and subsequently transfected using RNAi targeting ICAM-1 (siICAM-1#1) (n=3, I.I., invasion index, mean ± s.d., ***P<0.001). Scale bar 100μm. **C.** Immunoblot of ICAM-1 in hDF transfected with an empty vector (GFP) or ICAM-1 (ICAM-1-GFP). Immunoblot of GFP and tubulin as internal controls. **D.** Percentage of gel contraction by control (hDF-GFP) or HDF stimulated by LIF or overexpressing ICAM-1 (hDF-ICAM-1-GFP#A and #B) in presence or absence of a specific ICAM-1 blocking antibody (aICAM-1) (n=3 in triplicates, mean + s.d, ***p<0.001). **E.** Representative images of H&E staining of paraffin-embedded sections of SCC12 in response to control (hDF-GFP) or ICAM-1 overexpressing hDF (hDF-ICAM-1-GFP#A and #B) (n=33, I.I., invasion index, mean ± s.d., ***P<0.001). Scale bar 100μm. **F.** Representative images of H&E staining of paraffin-embedded sections of SCC12 in response to CAF sorted for ICAM-1 expression (n=3, I.I., invasion index, mean ± s.d., ***P<0.001). Scale bar 100μm.

ICAM-1 has been identified as an inflammatory responsive gene displaying increased expression in pathological tissues [21]. Therefore, we hypothesized that inflammation may induce development of a cancerous and proinvasive ECM *in vitro*. To verify this idea, hDF cells were grown in low serum media supplemented with pro-inflammatory cytokines known to play major roles in cancer development. All the tested pro-inflammatory cytokines, including TGFβ, LIF, TNFα, GCSF and IL6, induced a strong ICAM-1 expression in hDF (Figure 3A) and proinvasive activation of normal fibroblasts, which resulted in invasion of SCC12 cells in organotypic invasion assays (Figure 3B). Moreover, RNAi-mediated silencing of ICAM-1 blocked the proinvasive activity of inflammation-activated fibroblasts (Figures 3B and Supplementary Figure S3A). This result demonstrates that membrane-bound ICAM-1 expression in fibroblasts supports the inflammation-dependent extracellular matrix remodeling and may drive inflammation-dependent fibrosis leading to organ failure in multiple pathologies [30].

To investigate whether ICAM-1 is sufficient to support fibroblast-dependent matrix remodeling and proinvasive activities, hDF constitutively expressing high level of membrane-bound ICAM-1 were generated (Figure 3C). Their contractile and proinvasive capacities *in vitro* were assessed using two independent hDF cell lines (hDF-ICAM-1-GFP#A and #B). ICAM-1 expression resulted sufficient to induce both matrix contraction (Figure 3D) and proinvasive activities (Figure 3E). Addition of an ICAM-1 blocking antibody during matrix contraction strongly decreased the contractile capacity of the ICAM-1 overexpressing fibroblasts to a level comparable to that of the control parental cell (Figure 3D). It is well established that CAF consist of highly heterogeneous sub-populations within the tumor, a heterogeneity conserved during culture *in vitro* [31]. Accordingly, heterogeneity of CAF for membrane-bound ICAM-1 expression was confirmed by FACS cell sorting (Supplementary Figure S3B), which allowed to investigate the possible correlation between the contractile and proinvasive capacities of CAF subpopulations with the levels of ICAM-1 expression at the cell surface (Supplementary Figure S3C). It could also be demonstrated that the ability of collagen contraction by CAF depends on the level of ICAM-1 expression (Supplementary Figure S3D) and that ICAM-1 expressing cells acquire proinvasive capacities compared to low-ICAM-1 expressing CAF (Figure 3F). Membrane-bound ICAM-1 expression and association with CAF-marker expression in CAF was further investigated (Supplementary Figure S3E), which showed that CAF expressing high level of membrane-bound ICAM-1 also express high level of the CAF markers FAP1, αSMA and fibronectin, These data demonstrate that membrane-bound ICAM-1 is necessary and sufficient to promote proinvasive ECM remodeling by CAF, and that membrane-bound ICAM-1 may serve as a marker to identify the proinvasive stromal fibroblasts.

### Src kinases mediate ICAM-1-dependent regulation of CAF acto-myosin contractility

**Figure 4:**
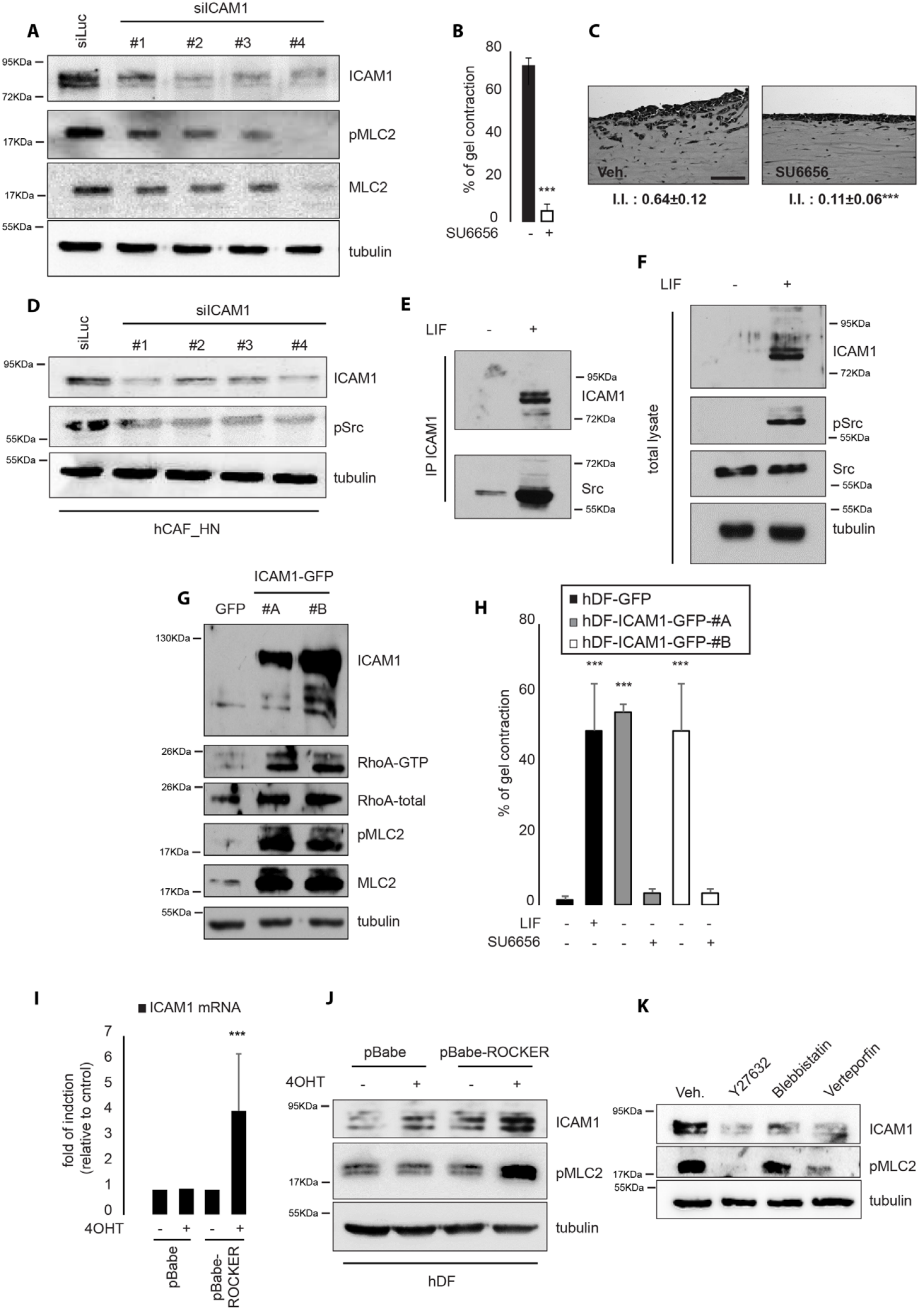
ICAM-1 regulates and is regulated by acto-myosin contractility **A.** Immunoblot of ICAM-1, pMLC2 and MLC2 in CAF following transfection of RNAi targeting ICAM-1 (siICAM-1#1, #2, #3 and #4). Tubulin as internal control. **B.** Percentage of gel contraction by CAF in presence or absence of SU6656. (n=3 in triplicates, mean + s.d, ***p<0.001). **C.** Representative images of H&E staining of paraffin-embedded sections of SCC12 in response to CAF in absence or presence of SU6656 (n=3, I.I., invasion index, mean ± s.d., ***P<0.001). Scale bar 100μm. **D.** Immunoblot of ICAM-1, pSrc in CAF following transfection of RNAi targeting ICAM-1 (siICAM-1#1, #2, #3 and #4). Tubulin shown as control. **E.** Immunoblot of ICAM-1 and Src in hDF stimulated by LIF following ICAM-1-specific immunoprecipitation. **F.** Immunoblot of total cell lysate from experiment shown in E. Immunoblot of ICAM-1 and pSrc in hDF stimulated by LIF. Tubulin and Src shown as control. **G.** Immunoblot of ICAM-1 and RhoA following RhoA-GTP pull down assay in hDF cell transfected with an empty (GFP) or ICAM-1-expressing vector (ICAM-1-GFP#A and #B). Immunoblot of pMLC2, MLC2 and total RhoA shown as controls. **H.** Percentage of gel contraction after 6 days by control (hDF-GFP) or ICAM-1-overexpressing hDF (hDF-ICAM-1-GFP#A and #B) in presence or absence of SU6656 (n=3 in triplicates, mean + s.d, ***p<0.001). **I.** Quantification of ICAM-1 mRNA levels in control or HDF overexpressing an active form of ROCK following 4OHT treatment (n=3 in triplicates, mean + s.d., ***p<0.001). **J.** Immunoblot of ICAM-1 in control or HDF overexpressing an active form of ROCK following 4OHT treatment. Immunoblot of pMLC2 and tubulin shown as controls. **K.** Immunoblot of ICAM-1 in hDF cell control (veh.) or treated with Y27632, bebblistatin or verteprofin. Immunoblot of pMLC2 and tubulin shown as controls.

Acto-myosin contractility is a key driver for CAF-dependent proinvasive ECM remodeling and its regulation by pro-inflammatory cues, such as IL6 family cytokines, requires cooperation between the JAK1 and ROCK kinases [10]. Thus, we speculated that membrane-bound ICAM-1 might drive proinvasive ECM remodeling by CAF via regulation of acto-myosin contractility and subsequently through MLC2 phosphorylation on Serine 19, which reflects MLC2 activation. Accordingly to this hypothesis, RNAi-mediated ablation of ICAM-1 expression was found to lead to a strong decrease of MLC2 phosphorylation (Figure 4A) subsequent to a reduced activity of the RhoA small GTPase upon ICAM-1 depletion (Supplementary Figure S4A). A strong correlation between ICAM-1 expression and the endogenous level of activated MLC2 was also established (Supplementary Figure S4B). In light of these data we confirmed that membrane-bound ICAM-1 regulates the onset of a proinvasive tumor microenvironment potential in CAF by regulation of the acto-myosin cytoskeleton contractility.

The molecular mechanisms underlying the ICAM-1-dependent MLC2 regulation in hCAF were then deciphered. Src family kinases have been linked to ICAM-1-dependent cell signaling [32] and to MLC2 regulation [33]. The potential role of the Src family kinases in CAF-dependent ECM remodeling remains, however, poorly studied. Whether the Src family kinases could play a role in CAF-dependent ECM remodeling and SCC12 collective invasion was thus investigated *in vitro* using a three-dimensional organotypic invasion assay. In our hands, addition of SU6656, a Src kinase family inhibitor, strongly impaired both gel contraction and SCC12 cell collective invasion (Figure 4B and 4C). Moreover, CAF presented high levels of endogenous Src-activated phosphotyrosin 416. However, RNAi-mediated ablation of ICAM-1 expression, or addition of SU6656, induced a strong decrease of 416 tyrosin residue phosphorylation (Figure 4D and Supplementary Figure S4C). Accordingly, the crucial role for Src kinases during proinvasive fibroblast activation was demonstrated. Indeed, LIF-mediated Src phosphorylation at Y416 (Supplementary Figure S4D) was mandatory for LIF-induced ECM remodeling and also SCC12 cell invasion (Supplementary Figures S4E and S4F). Co-immunoprecipitation assays demonstrated that LIF stimulation induces binding of Src kinases to ICAM-1 in hDF, (Figure 4E and 4F), which further reinforces the hypothesis that ICAM-1 mediates fibroblast acto-myosin contractility through a Src-dependent regulation of MLC2 activity. Finally, we confirmed that ICAM-1 overexpression is sufficient to promote activation of the RhoA/ROCK/MLC2 signaling pathway activation. Indeed, forced expression of ICAM-1 in hDF triggered an increased RhoA-GTP bound state together with an increased activity and expression of MLC2 protein (Figure 4G). Accordingly, the ICAM-1-overexpressing hDF displayed an increased contractility capacity that was abolished in presence of SU6656 (Figure 4H). On the other hand, inhibition of Src activity in hCAF resulted in decreased ICAM-1 expression and reduction of both MLC2 expression and activity (Supplementary Figure S4G). These data therefore demonstrate that membrane-bound ICAM-1 governs the onset of a proinvasive ECM remodeling through a Src/RhoA/ROCK/MLC2 signaling pathway. Interestingly, in CAF, inhibition of either activity or expression of JAK, a kinase family regulated by the IL6 family cytokines such as LIF, impaired ICAM-1 expression (Supplementary Figures S4H and S4I). Having demonstrated that JAK and ROCK signaling pathways cooperate to control acto-myosin contractility [10], we investigated whether cytoskeleton contractility in CAF could regulate ICAM-1 expression. Figure 4I and 4J show that forced expression of an active form of ROCK (ROCK-ER) [34] following 4-hydroxytamoxifen (4OHT) treatment is sufficient to increase *ICAM-1* expression at mRNA and protein levels, respectively. Moreover, inhibition of the mechano-responsive signaling pathway, which includes ROCK/MLCK and the YAP-TEAD interaction, using the Y27632, bebblistatin and verteporfin chemical compounds, respectively, reduced the ICAM-1 protein content in CAF (Figure 4K). Taken together, these data demonstrate that membrane-bound ICAM-1 regulates, and is regulated, by acto-myosin contractility, which attributes a central role to ICAM-1 in regulation of actin cytoskeleton contractility in the stroma fibroblasts.

### ICAM-1 expression in tumor stroma correlates with the presence of invasive cohorts of tumor cells in human head and neck carcinoma

**Figure 5:**
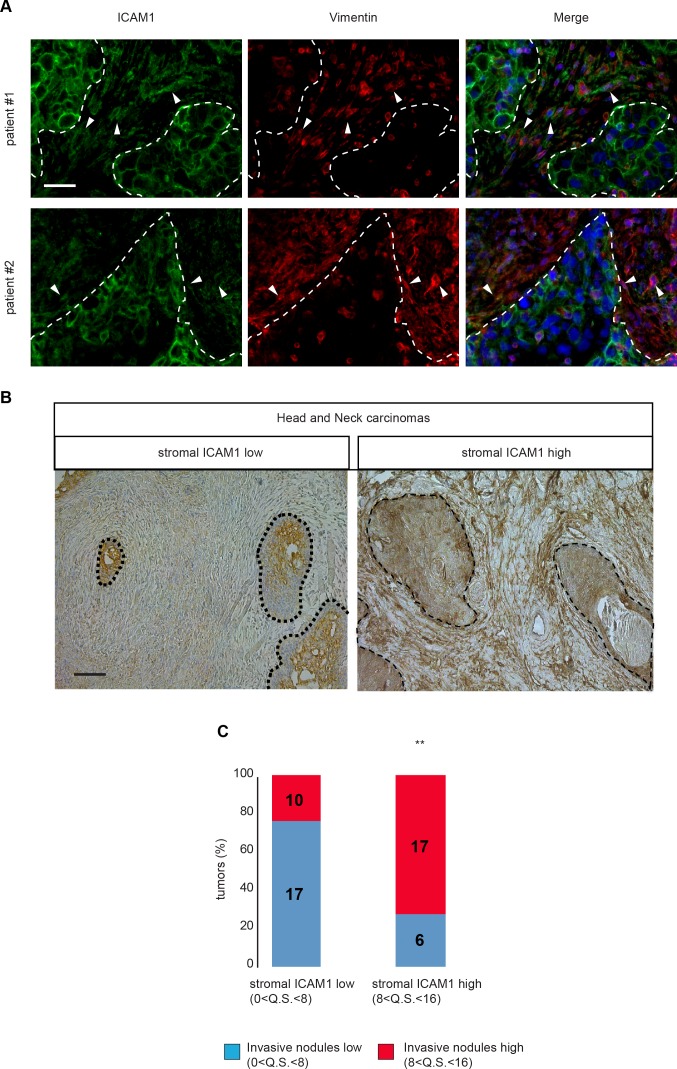
ICAM-1 is overexpressed in head and neck cancer stroma **A.** Immunofluorescence of ICAM-1 (green) and vimentin (red) in representative cancer biopsies from two distinct patients with head and neck carcinoma. **B.** ICAM-1 immunohistological staining in human head and neck (n=50) carcinomas. Left panel shows low ICAM-1 detection and right panel shows high ICAM-1 detection. Scale bar, 100 μm. **C.** Plot of mean quick score quantification for ICAM-1 specific decoration in tumor stroma from 48 head and neck carcinoma samples relative to the mean quick score for presence of invasive carcinoma cell nodules.

Overexpression of ICAM-1 in cancer tissues, both in tumor cells and stroma, has previously been reported. Indeed, ICAM-1 is up-regulated in CAF associated with colorectal cancer [27], but no information was so far available on possible functional consequences. Because our *in vitro* results suggest a novel role for ICAM-1 in tumor ECM remodeling and onset of proinvasive tumor stroma, expression of ICAM-1 was investigated in invasive tumor cell clusters in human head and neck carcinomas. Interestingly, ICAM-1 was found in the tumoral stroma and co-localized with vimentin, a fibroblast marker (Figure 5A). Analysis of ICAM-1 expression level in 48 human head and neck carcinomas, using the quick score method, was consistent with the quick score for the presence of the invasive cohorts of tumor cells observed in the tumor samples (Figures 5B and 5C and Supplementary Figure S5A). In conclusion, association of high levels of ICAM-1 with presence of invasive cohorts of tumor cells in human carcinomas validates our *in vitro* observations, and further indicates that ICAM-1 detection in tumor stroma may serve as a diagnostic tool to define the proinvasive potential of the tumor microenvironment.

## DISCUSSION

**Figure 6:**
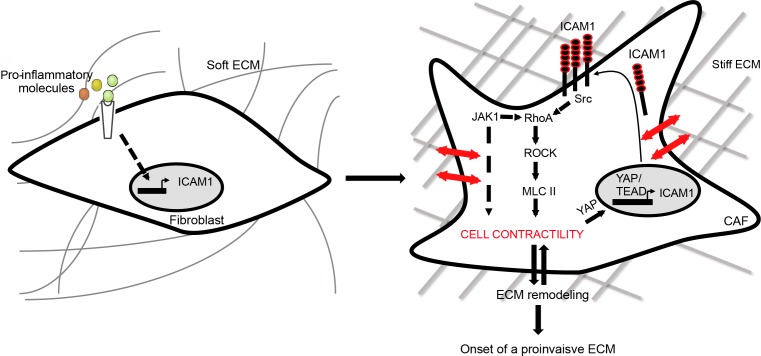
ICAM-1 mediates inflammation-dependent onset of a proinvasive ECM remodeling Pro-inflammatory cues from the tumor microenvironment trigger ICAM-1 expression in fibroblasts, which leads to proinvasive ECM remodeling. In activated fibroblasts, membrane-bound ICAM-1 expression controls cellular contractility through regulation of a Src/RhoA/ROCK/MLC2-dependent signaling. In return, acto-myosin contractility in CAF regulates expression of membrane-bound ICAM-1 at the cell surface. In CAF, ICAM-1 acts as a crucial hub to sustain acto-myosin contractility and matrix remodeling of tumor stroma.

We have identified a novel role for *ICAM-1*, an inflammatory responsive gene, in the establishment of a protumorigenic tumor stroma. In reaction to inflammatory signaling cues, fibroblasts populating the tumor stroma are activated, which induces ICAM-1 expression at the cell surface. As a consequence, activated fibroblasts promote the onset of proinvasive ECM remodeling leading to tumor cell invasion (Figure 6).

The pro-inflammatory cytokine LIF induces [9] and sustains [28] the proinvasive capacity of stromal fibroblasts through the constitutive activation of the JAK1/STAT3 signaling pathway [10]. In this study, we demonstrate that LIF supports the transcriptomic signature of fibroblasts long-term activated by TGFβ, a well-known CAF activator both *in vitro* and *in vivo* [7]. In addition, we show that LIF-responsive genes *in vitro* are those regulated in CAF, which validates the essential role of LIF in CAF activation during tumor progression.

Using three-dimensional phenotypic contraction assays, following RNAi-mediated knock-down expression of 50 LIF-responsive genes, we identified four genes (ICAM-1, DBC1, HRH1 and BCL3) consistently crucial for activated-fibroblast contractility. While DBC1, also called BRINP1 (Bone Morphogenic Protein/Retinoic Acid Inducible Neural-Specific 1), a cell proliferation and cell death regulator, has never been linked to cell contractility, which deserves investigation, HRH1 (Histamine Receptor 1) was shown to promote human lung fibroblast collagen lattice contraction *in vitro* [35]. Interestingly, RNAi-mediated ablation of BCL3 (B-cell CLL/Lymphoma 3) expression in activated fibroblasts, leads to a strong decrease of ICAM-1 expression both in LIF-activated fibroblasts and in CAF (data not shown). BCL3 is a transcriptional co-activator of NF-κB transcription factor that mediates TNFα-dependent ICAM-1 expression [21]. Because we consider that the *in vitro* three-dimensional collagen lattices contraction assay is a powerful tool to unveil new genes or signaling pathways that regulate cell contractility [10, 28, 29], such a screen was used to disclose a novel role for ICAM-1 during tumor progression.

Pro-inflammatory cytokines production is a key characteristic of tumor microenvironments [36–38] and cytokine signaling contributes to the establishment of a proinvasive ECM [9, 10]. The central role of the membrane-bound ICAM-1 that we have unveiled in the cytokine-dependent regulation of RhoA/ROCK/MLC2 acto-myosin cytoskeleton contractility in CAF is reinforced by the function that this protein plays in endothelial cells during leukocytes transendothelial migration [39, 40]. Also the fact that membrane-bound ICAM-1 regulates the Src kinases activity, which controls the RhoA/ROCK/MLC2 signaling pathway [32], is in accordance with previous data suggesting a role for Src in fibroblast contractility [41] and during kidney and lung fibrosis [42, 43]. Multiple cytokines signaling converge to ICAM-1 to promote and sustain proinvasive matrix remodeling, which makes membrane-bound ICAM-1 a potential target for therapeutic protocols for patients suffering from aggressive carcinoma. Indeed we demonstrate that membrane-bound ICAM-1 is overexpressed in CAF isolated from head and neck, lung and breast cancers, which strongly suggests that in such cells ICAM-1 plays a role consistent with the functions we unveiled *in vitro*. Because the need for specific CAF sub-population identification is compelling [7, 31], detection of ICAM-1 in the tumor stroma, coupled with a specific fibroblast marker might constitute an interesting biomarker for clinical evaluation of tumor stroma proinvasiveness. ICAM-1 belongs to a family of five members (ICAM-1 to ICAM-5) that share some, but not all, structural similarities and disclose diverse patterns of expression and downstream effector behaviors [44]. For instance, ICAM-1 blocking antibodies do not interfere with ICAM-2-dependent leukocyte adhesion to endothelial monolayers [45]. Moreover, ICAM-2 is not regulated by inflammatory cues [46]. ICAM-3 shows a different binding property to LFA-1 [47, 48]. All together, these data suggest that ICAM-1 present unique features and biological activities, such as matrix remodeling in CAF. Accordingly, our pan-genomic data show that ICAM-1 is the only family member to be induced by LIF and TGFβ in hDF cells, and specific knock down expression of ICAM-1 drastically blocks CAF contractility, which indicates that in the context of proinvasive matrix remodeling by CAF no compensation mechanisms are provided by the other ICAM family members. In contrast, little is known about the source of sICAM-1 that is found to correlate with tumor stage and metastasis development in sera of patients with carcinoma [49–54]. It is proposed that sICAM-1 acts as a de-adhesive molecule that triggers cancer cell migration: investigating the potential secretion of sICAM-1 by CAF would therefore be of general interest in cancer biology.

Interestingly, our results demonstrate that ICAM-1 regulates fibroblast contractility. In CAF, inhibition of ROCK, MLCK and the YAP-TEAD complex drastically downregulates ICAM-1 expression. Accordingly, forced expression of an active form of ROCK is sufficient to trigger ICAM-1 expression in fibroblasts. These observations are consistent with the fact that, in endothelial cells, application of mechanical forces to ICAM-1 clusters induces a Rho GEF 12-dependent RhoA activation [55], and ICAM-1 clustering at the cell surface is sufficient to promote traction forces [56]. Moreover, it has been suggested that a positive feedback signaling between acto-myosin contractility and matrix stiffness may sustain CAF contractility in the tumor stroma [41]. Based on our results, one can speculate that ICAM-1 acts as a central hub that coordinates a signaling loop between acto-myosin contractility and matrix stiffening that sustains the onset of a proinvasive tumor microenvironment (Figure 6). However, how ICAM-1 could sense matrix stiffness remains to be elucidated. Integrins are considered the main mechanoreceptors able to transduce out-side-in signaling in response to matrix stiffness [57]. In CAF, α3β1 and α5β1 integrins are involved in matrix remodeling and proinvasive behavior through the regulation of cell contractility and matrix remodeling [58–60]. We can thus speculate that membrane-bound ICAM-1, similarly to its role of tyrosine kinase co-receptor [61], may interact with integrins to transduce the out-side-in signaling. On the other hand, ICAM-1 interacts within the first immunoglobulin domain of fibrinogen to support the Src-dependent mitogenic activity in B cells [15, 62]. In light of this data, it is tempting to speculate that ICAM-1 may bind to extracellular fibrinogen to promote acto-myosin contractility in CAF.

In conclusion, we identify membrane-bound ICAM-1 as a major regulator of proinvasive CAF activity in head and neck carcinoma, but a similar role could also be played in lung and breast carcinomas. We demonstrate that membrane-bound ICAM-1 promotes inflammation-dependent extracellular matrix remodeling, which leads to tumor cell dissemination. Membrane-bound ICAM-1 is overexpressed in CAF and acts as a crucial hub to sustain acto-myosin contractility and matrix remodeling in tumor stroma. Therefore our data suggest that inhibition of membrane-bound ICAM-1 function using ICAM-1 specific blocking antibodies might constitute an interesting possibility to counteract tumor cell invasion and dissemination.

## MATERIALS AND METHODS

### Cell culture

Human primary Dermal Fibroblasts (hDF) and human HEK293 Phoenix cells were maintained in DMEM supplemented with 10% FCS (fetal calf serum). Human Carcinoma-Associated Fibroblasts (CAF) isolated from patients with head and neck, lung and breast cancers were cultured in DMEM supplemented with 10% FCS and insulin-transferrin-selenium (#41400-045; Invitrogen, Carlsbad, CA). SCC12 cells were cultured in FAD media, as described in Gaggioli et al [59].

Long-term LIF and TGFβ1-activated fibroblasts (hDF_LIF and hDF_TGFβ1) have been performed in DMEM supplemented with 0.5% FCS containing 2ng/ml final concentration of human recombinant proteins for seven days. Next, activated-hDF was cultured for 7 days in 0.5% FCS media prior to experiments.

### Cytokines and neutralizing antibodies and inhibitors

TGFβ1 was purchased from Peprotech (#100-21, Peprotech, Rocky Hill, NJ) and was used at 2 ng/ml; recombinant human GCSF (#300-23) and IL-6 (#200-06) were purchased from Peprotech and were used at 10ng/mL, recombinant human LIF was purchased from Millipore (#LIF1005, Millipore, Billerica, MA), and was used at a concentration of 2 ng/ml. Recombinant TNF alpha was produced in E Coli and purified under native conditions using an N-terminal 6-his tag. ICAM-1 neutralizing antibody (#BBA3, R&D, Minneapolis, MN) was used at 10 μg/ml. The following inhibitors were used in this study: Ruxolitinib (#1598, Axon medchem, Groningen, The netherlands) at 10μM, CYT387 (#S2219, Selleckchem, Huston, TX) at 10μM Y27632 (#1254, Tocris bioscience, Ellisville, MO) at 10μM, Blebbistatin (#B0560, Sigma, Saint Louis, MO) at 10μM, SU6656 (#572635, CalbioChem, Los Angeles, CA) at 10μM and Verteprofin (#SML0534, Sigma, Saint Louis, MO) at 4μg/mL.

### RNAi transfections

Cells were plated at 60% confluence and subjected to transfection the following day using Dharmafect 3 (#T-2002-02; Dharmacon, inc., Lafayette, CO) at 20nM final concentration of RNAi. RNAi sequences are listed in Supplementary Table S2.

### Organotypic invasion assays and matrix remodeling assay

In organotypic invasion assays, 5.10^5^ fibroblasts were embedded in 1ml of matrix gel, made of collagen I and Matrigel, yielding a final collagen concentration of 4.6 mg.ml^−1^ and a final Matrigel concentration of approximately 2.2 mg.ml^−1^. After 1h at 37°C, matrix gel were overlaid with 5.10^5^ SCC12 cells and lifted at the cells-air interface 24h later. After 5 days, organotypic cultures were fixed, embedded in paraffin block, sectioned and stained for invasion index quantification using ImageJ [63]. For gel contraction assay, 25.10^3^ cells were embedded in 100μl of matrix gel [29] and seeded in triplicate into 96 wells plate. After 1h at 37°C, matrix gels were overlaid with 100μl of 0.5% FCS medium (with indicated cytokines or inhibitors) and changed every two days. At day 6 the relative diameter of the well and the gel were measured using ImageJ. The percentage of gel contraction was calculated using the formula 100 x (well diameter – gel diameter) / well diameter.

### Neutralizing antibody method

Neutralizing antibody against ICAM-1 (10μg/mL) was incubated one hour with trypsinized fibroblasts at 37°C before being used for matrix remodeling assay or organotypic cultures. Neutralizing antibody against LIF (10μg/mL) was incubated one hour with media before fibroblasts stimulation.

### Antibodies

Antibodies against STAT3 (#9139; 1/1000), pY705-STAT3 (#9145; 1/1000), JAK1 (#3332; 1/500), pY1022/1023-JAK1 (#3331; 1/200), MLC2 (#3672; 1/500), pThr18/19-MLC2 (#3674; 1/500) Src (#2109; 1/2000), pTyr416-Src (#2101; 1/500), vimentin (#5741) were purchased from Cell Signaling (Cell SignalingTechnology, Beverly MA), α-tubulin from sigma (T4026, Sigma, Saint Louis, MO; 1/5000) ICAM-1 (#sc-8439; 1/1000), RhoA (#sc-418; 1/500) from Santa Cruz Biotechnology (Santa Cruz Biotechnology, Santa Cruz, CA).

### Western blot and coimmunoprecipitation analysis

Western blot analysis was performed as previously described [9]. For coimmunoprecipitation analysis, cells were lysed on ice in modified RIPA buffer (50mM Tris pH 7.4 150 mM NaCl, 1%NP-40, 0.1% SDS, 0.5% SD Dexoxycholate, 5mM NaF, 2.5 mM Nappi, and protease inhibitor (#04693159001, Roche) for 30 minutes and isolated by centrifugation (15 min, 10000g, 4°C). Supernatants were precleared during 1 hour at 4°C with Magna CHIP protein G bead (#16-662, Millipore) and normal mouse IgG (#sc-2025, Santa Cruz technology) and the cleared lysate incubated with primary antibody or IgG overnight at 4°C. Immune complexes were captured by adding 35μl of protein G magnetic beads, rotated for 1h at 4°C and washed three times with lysis buffer (without SDS, Sodium Deoxycholate and protease inhibitors). Immunoprecipitation products were separated by SDS-PAGE.

### Microarrays analysis

Total RNA of hDFs stimulated by LIF, TGFβ or TGFβ + αLif mAb was extracted using the RNeasy kit (Qiagen, Hilden, Germany). The integrity of the RNA was assessed using an Agilent BioAnalyzer 2100 (Agilent Technologies). RNA samples were then labeled and hybridized on 8×60K high density SurePrint G3 gene expression human Agilent microarrays following the manufacturer's instructions. Two biological replicates were performed for each experimental condition. The microarray experimental data were deposited in the NCBI GEO under the serial record number GSE81996 (short term response) and GSE81997 (long term response).

The data were quantile normalized using the Bioconductor package limma [64]. Means of ratios from all comparisons were calculated and the moderated t-statistic of the limma package provided the per gene *P* values. The Benjamini-Hochberg procedure was used to control the experiment-wise false discovery rate (FDR) from multiple testing procedures. Differentially expressed genes were selected based on an adjusted p-value below 0.05 and an absolute log2 (fold change) >0.7.

### RT-qPCR analysis

RNA isolation was performed using RNeasy Mini kit (#217004, Qiagen, Turnberry Ln, Valencia, CA) according to the manufacturer's instructions. Reverse Transcription of 500ng RNA by Superscript II reverse transcriptase (#18064-014, Invitrogen, Carlsbad, CA) was followed by Real time PCR using Fast SYBR Green Master Mix (#18064-014; Applied Biosystems, Foster City, CA) and performed on a Step One Plus Real-Time PCR system (Applied Biosystems, Foster City, CA).

Primers sequences are listed in Supplementary Table 2:

Relative expression of the respective gene was determined after normalization to GAPDH and calculated with the following formula: relative expression level = 2ddCT

### Statistical analysis

Student's t test was performed for statistical analysis of invasion assay, gel contraction assay, and qPCR results. *** indicates p<0.001, ** indicates p<0.01, * indicates p<0.05. Error bars are + standard deviation (+ s.d.). Pearson's correlation coefficient was used to assess the relationship between ICAM-1 and invasion quick score within human samples.

### Immunohistochemical staining and quantification methods

Fifty head and neck tumor biopsies were fixed (3.7% formaldehyde in PBS) for 4 h and transferred to 70% ethanol (24 h), embedded in paraffin wax and sectioned at 7 μm. After deparaffination, microwave antigen retrieval was performed in Na-citrate buffer (10mM, pH6; 5min at 900W, 10min at 150W and 30 min at room temperature). Sections were washed three times in PBS (5min per wash) before endogenous peroxidase activity was blocked in 1% H2O2 for 10 min and washed 3 times in PBS. After incubation in blocking buffer for two hours (10% serum (S-5000, S-1000; Vector, Burlingame, CA); 0.3% Triton X100 in PBS), sections were incubated with primary antibody diluted 1:50 in blocking buffer overnight at 4°C. After three washes in PBS, sections were incubated with biotinylated secondary antibody (#BA-1000 Vector, Burlingame, CA) diluted 1:400 in PBS for 30 min and washed 3 times in PBS. Samples were then processed using Vectastain ABC kit (#PK4001, Vector, Burlingame, CA) and DAB peroxidase substrate kit (#SK4100, Vector, Burlingame, CA) according to manufacturer's instructions. Sections were next counterstained with hematoxylin for 5 sec, rinsed in water, blued 10 sec in 0,08% ammonia water, dehydrated, cleared, and mounted with cover clips. Two authors, blinded to each other's assessment, scored the slides using the Quick Score method to determine ICAM-1 status within the tumor bulk and stroma.

For immunofluorescence staining, after deparaffination and microwave antigen retrieval, sections were washed three times in PBS (5min per wash) before incubation in blocking buffer (3% BSA #A7030, Sigma; 0.2% Triton X100 in PBS) for 1 hour. Sections were incubated with primary antibodies diluted 1:50 in blocking buffer overnight at 4°C. After three washes in PBS, sections were incubated with secondary antibody conjugated to Alexa 488 (#A21202, Life Technology) or Alexa 594 (#A21207, Life Technology) diluted 1:400 in PBS for 1 hour and washed 2 times in PBS, stained with DAPI (2μg/mL) for 5min, rinsed in water and coverslips mounted onto glass slides using mounting media (#TA-030-FM, Thermo).

### Plasmids constructs

ICAM-1 was cloned in pEGFPN1 vector between BamH1 and Age1 sites after PCR amplification using following primers: forward : CGCGGGGGATCCGCCACCATGGCTCCCAGCAGCCCCCG reverse : CGCGGGACCGGTGTGGGAGGCGTGGCTTGTGTG; The ICAM-1-GFP insert was retrieved by cutting with BamH1 and Hpa1 restriction enzymes and subcloned in the pBABE puro vector between the BAMH1/SnaB1 sites, generating the pBABE-ICAM-1-GFP Puro construct. The same operation was realized with the GFP gene alone to make the pBABE-GFP Puro control vector.

### Production of recombinant retroviruses

Phoenix cells were transiently transfected with 1μg of the previously described vectors using calcium phosphate mediated transfection using classical procedures. Six hours after transfection, cells were washed with PBS and complete media was added. The day after, media was replaced by a heat-inactivated serum medium and cells were moved to 32°C for 24 hours. Forty eight hours post transfection clarified supernatents (retroviral particles) were collected and used to infect either hDF. Retrovirus infection was performed in the presence of 5ug/ml polybrene. Stably transduced cells were selected with 5ug/ml puromycin.

### Flow cytometry

For ICAM-1 labelling, fibroblasts were trypsinized, resuspended in DMEM 10% FCS and washed in PBS. 1 × 10^6^ cells/100 μL were blocked in PBS containing 2% FCS and 0,5mM EDTA (FACS buffer) for 1 hour at 4°C. PE-conjugated antibody for ICAM-1 (#353105, BioLegend) or normal mouse IgG (#400113, BioLegend) were add at 1/100 dilution in blocking buffer during 1 hour at 4°C. Cells were washed three times in PBS and resuspended in FACS buffer at 1 × 10^6^ cells/100 μL. Analysis was performed on a FacsCanto flow cytometer (Becton Dickinson) with BD diva software. Cell sorting was performed on FacsAria 3 (Becton Dickinson). Cells collected were cultured for 7 days in 0.5% FCS containing 1% of Penicilin-Streptomycin (#15140-122, Gibco) and 0,5% of Fongizone (#15290-018, Gibco) prior to experiments.

### Conditionned media preparation

Cancer cells were grown to confluence, washed twice with PBS and then incubated in serum-free medium at 37°C. After 48 hours, conditioned media (CM) were collected, centrifuged at 5000g for 5 min to remove cell debris and the supernatant stored at −80°C.

### Pulldown RhoA activity

Fibroblasts were grown to 80% of confluence and lysed on ice in lysis buffer (50mM Tris pH 7.5, 500mM NaCl, 0.1% SDS, 1% triton, 0.5mM MgCl2 and protease inhibitor (#04693159001, Roche)) for 5 minutes and isolated by centrifugation (5 min, 10000g, 4°C). Equal concentration and volume of each sample were used and 30μg of GST-RBD beads was added. Samples were incubated at 4°C with rotation for 30 minutes. Beads were washed 4 times with lysis buffer (150mM NaCl, without SDS) and resuspended in Laemli buffer. Samples were loaded on sodium dodecyl sulfate-polyacrylamide gel electrophoresis as described.

## SUPPLEMENTARY FIGURES AND TABLES














