# Supplementary material for: Membrane-bound ICAM-1 contributes to the onset of proinvasive tumor stroma by controlling acto-myosin contractility in carcinoma-associated fibroblasts
Source: Oncotarget. 2016 Nov 25;8(1):1304–20. doi: 10.18632/oncotarget.13610 (PMC5352056; doi:10.18632/oncotarget.13610)
Supplement: Supplementary file 1 [file oncotarget-08-1304-s001.pdf]

Membrane-bound ICAM-1 contributes to the onset of proinvasive tumor stroma by controlling acto-myosin contractility in carcinoma-associated fibroblasts

SUPPLEMENTARY FIGURES AND TABLES

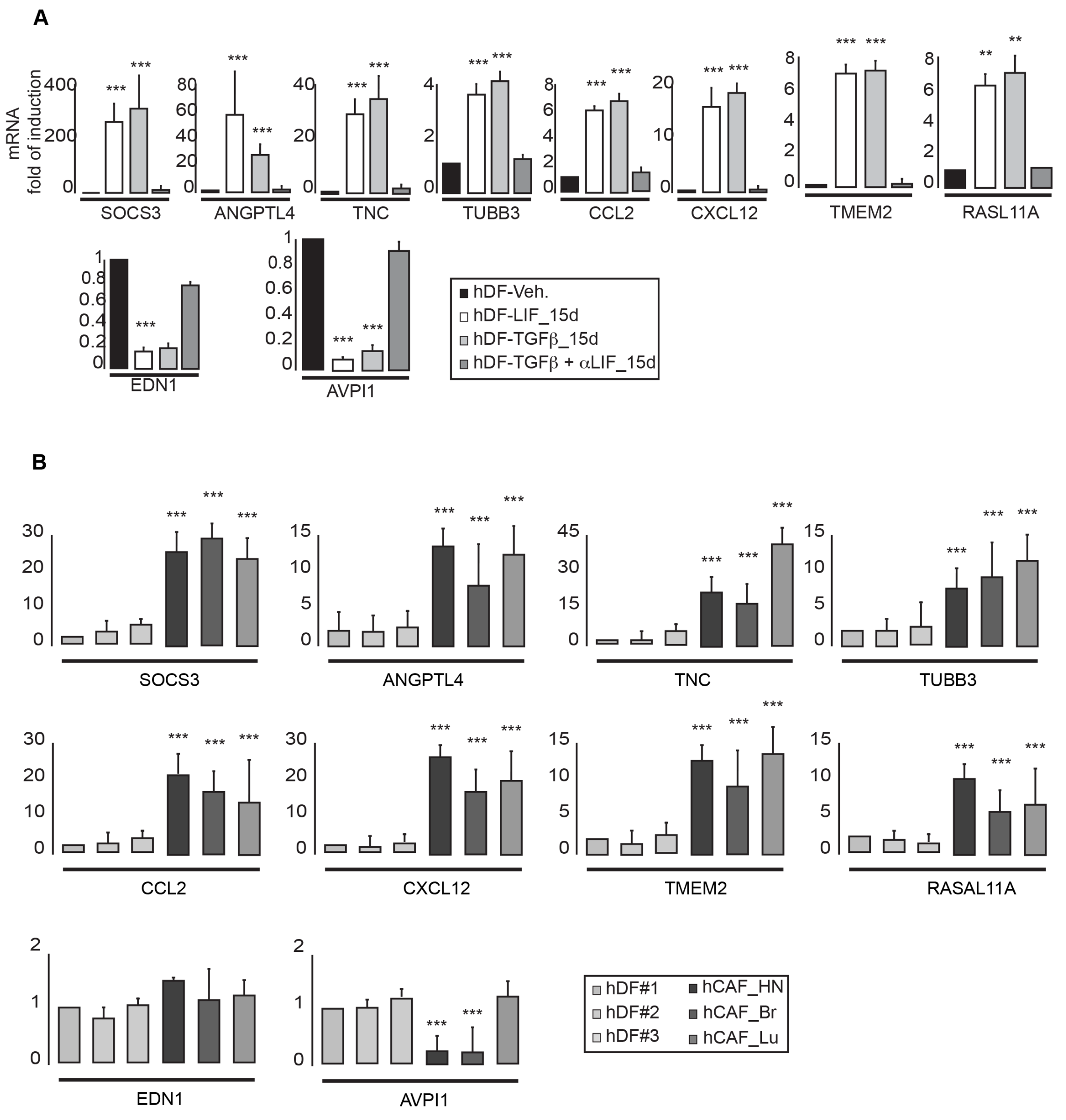

Supplementary Figure S1: A. Quantification of mRNA level in hDF following long-term LIF or TGFβ1 stimulation in presene or absence of LIF blocking antibody (n=3 in triplicates, mean + s.d., ***p<0.001). **B.** Quantification of mRNA level in three hDF strains and three hCAF isolated from head and neck, lung and breast carcinomas (n=3 in triplicates, mean + s.d., ***p<0.001).

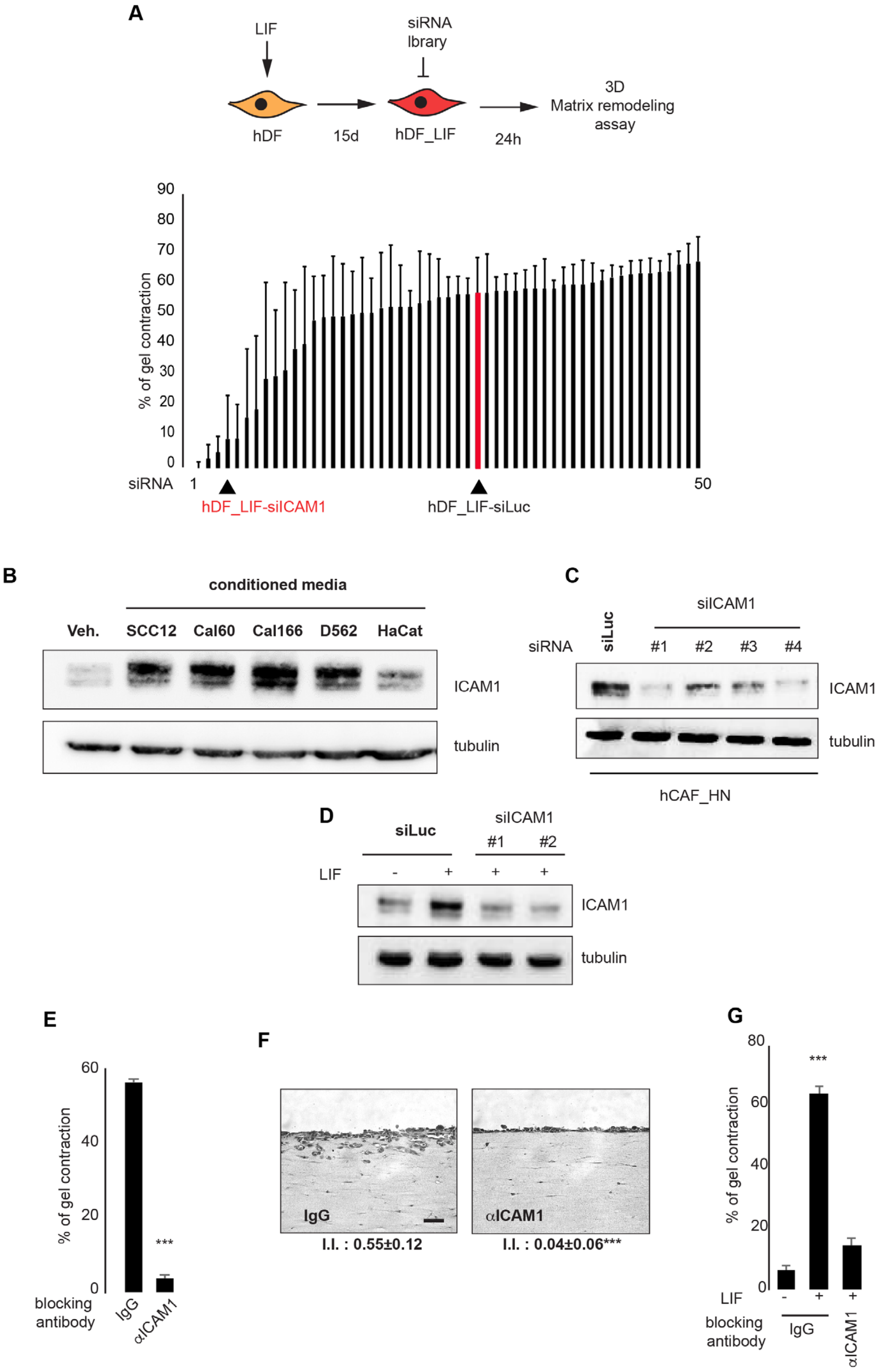

Supplementary Figure S2: A. Percentage of gel contraction after 6 days by hDF following long-term LIF stimulation and subsequent RNAi transfection (left panel, n=2 in triplicates). Schematic representation of experimental condition (top panel). **B.** Immunoblot of ICAM-1 in a panel of human SCC cells (SCC12, Cal60, Cal166, Detroit 562) and immortalized keratinocyte (HaCat). Immunoblot of tubulin shown as control. **C.** Immunoblot of ICAM-1 in CAF following transfection of RNAi targeting ICAM-1 (ICAM-1#1, #2, #3 and #4). Immunoblot of tubulin shown as control. **D.** Immunoblot of ICAM-1 in hDF in control or activated by LIF following transfection of RNAi targeting ICAM-1 (ICAM-1#1 and #2). Immunoblot of tubulin shown as control. **E.** Percentage of gel contraction by CAF in presence of IgG control or ICAM-1 blocking antibody (αICAM-1) (n=3 in triplicates, mean + s.d., ***$p<0.001$). **F.** Representative images of H&E colouration of paraffin-embedded sections of SCC12 in response to CAF in control (IgG) or in presence of ICAM-1 blocking antibody (αICAM-1) (n=3, I.I., invasion index, mean ± s.d., ***$P<0.001$). Scale bar 100μm. **G.** Percentage of gel contraction by hDF in control or LIF stimulated in presence of IgG control or ICAM-1 blocking antibody (αICAM-1) (n=3 in triplicates, mean + s.d., ***$p<0.001$).

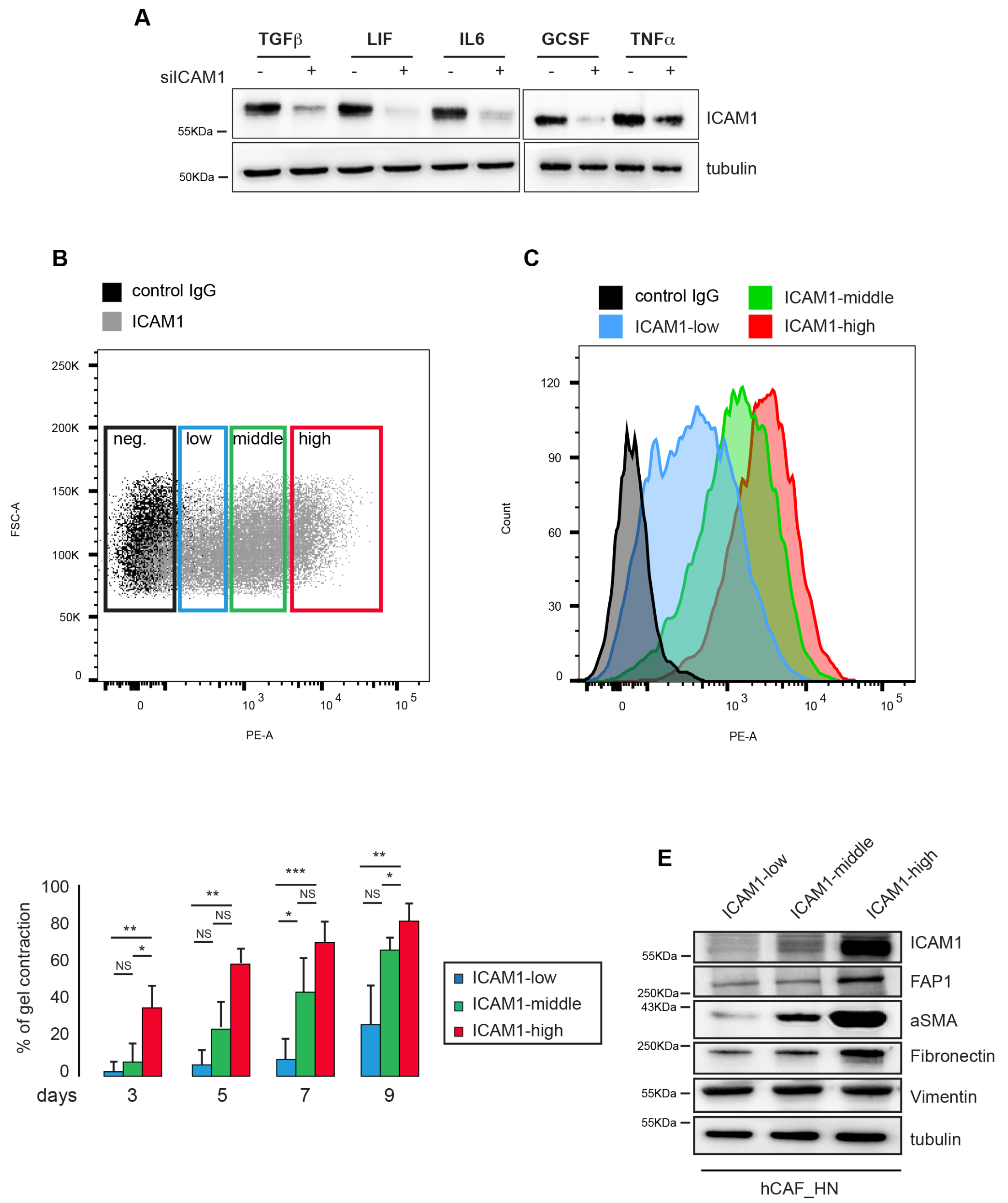

Supplementary Figure S3: A. Immunoblot of ICAM-1 in hDF transfected by RNAi targeting ICAM-1 and subsequently stimulated by inflammatory cytokines for 48h. Immunoblot of tubulin shown as control. **B.** Representative image of fluorescence intensity of ICAM-1 staining in CAF before FACS cells sorting. Boxes represent the FACS cells sorting gates for ICAM-1 staining intensity. **C.** Representative image of fluorescence intensity of ICAM-1 staining in CAF after FACS cells sorting for low, middle and high ICAM-1 staining intensity. **D.** Percentage of gel contraction by CAF after FACS cells sorting for ICAM-1 staining intensity (n=3 in triplicate, mean + s.d. NS for not significant, ***$p<0.001$, **$p<0.01$ and *$p<0.05$). **E.** Immunoblot of ICAM-1, FAP1, aSMA, fibronectin and vimentin in CAF expressing low, middle or high level of ICAM-1. Immunoblot of tubulin shown as control.

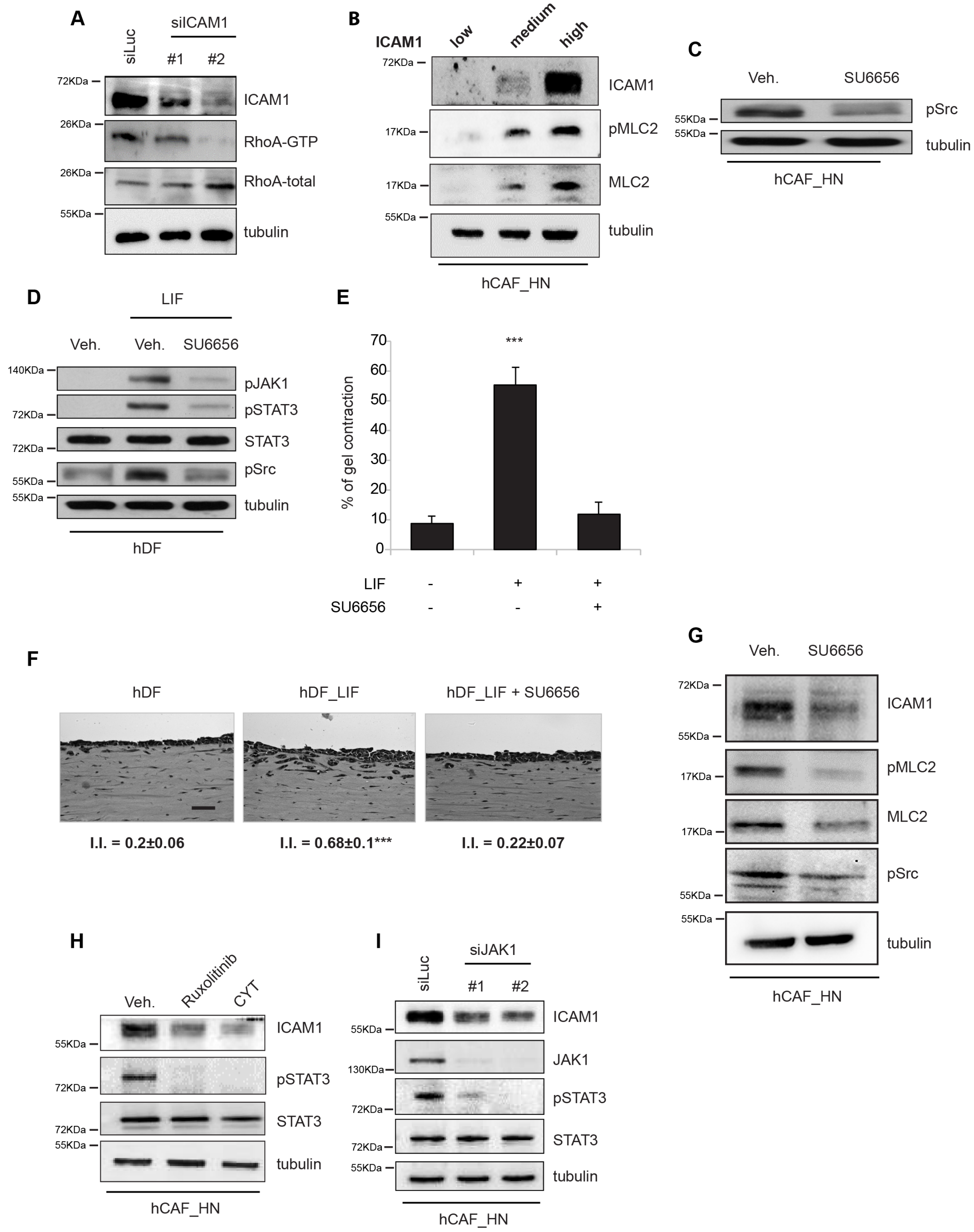

Supplementary Figure S4: A. Immunoblot of active RhoA (RhoA-GTP) in CAF following transfection of RNAi targeting ICAM-1 (siICAM-1#1 and #2). Immunoblot of ICAM-1, RhoA and tubulin shown as controls. **B.** Immunoblot of pMLC2 and MLC2 in CAF following FACS cells sorting for ICAM-1 staining intensity. Immunoblot of ICAM-1 and tubulin shown as controls. **C.** Immunoblot of pSrc in CAF in presence or absence of SU6656. Immunoblot of tubulin shown as control. **D.** Immunoblot of pJAK1, pSTAT3 and pSrc in hDF following LIF stimulation for 48h in absence or presence of SU6656. Immunoblot of STAT3 and tubulin shown as controls. **E.** Percentage of gel contraction by hDF stimulated by LIF in absence or presence of SU6656 (n=3 in triplicate, mean + s.d., ***p<0.001). **F.** Representative images of H&E colouration of paraffin-embedded sections of SCC12 in response to hDF in control or stimulated by LIF in absence or presence of SU6656 (n=3, I.I., invasion index, mean ± s.d., ***P<0.001). Scale bar 100μm. **G.** Immunoblot of ICAM-1, pMLC2 and MLC2 in hCAF following SU6656 treatment for 48h. Tubulin and pSrc shown as controls. **H.** Immunoblot of ICAM-1 and pSTAT3 in CAF in absence or presence of Ruxolitinib or CYT385 inhibitors. Immunoblot of STAT3 and tubulin shown as controls. **I.** Immunoblot of ICAM-1 and pSTAT3 in CAF following transfection of RNAi targeting JAK1 (siJAK1#1 and #2). Immunoblot of JAk1, STAT3 and tubulin shown as controls.

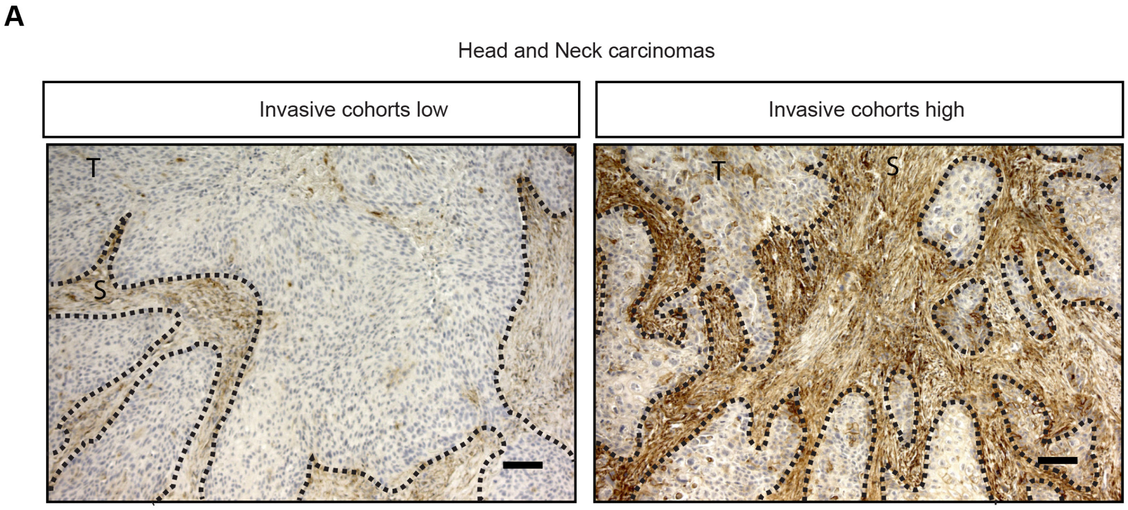

Supplementary Figure S5: A. ICAM-1 immunohistological staining in human head and neck carcinomas. Left panel shows a representative sample of low invasive nodules detection (0<Q.S.<8) and right panel shows a representative sample of high invasive nodules detection (8<Q.S.<16). Scale bar, 100 µm.

Supplementary Table S1A: 50 selected genes list and screens raw data. **A.** List of the 50 pan-genomic LIF-dependent genes selected. **B.** Raw data from initiation three-dimension contraction screen assays with hDF short term LIF activated and transfected by the 50 RNAi and subsequently transferred in collagen gel (n=2 in triplicate). **C.** Raw data from maintenance three-dimension contraction screen assays with hDF long term LIF activated and transfected by the 50 RNAi and subsequently transferred in collagen gel (n=2 in triplicate). **D.** Raw data from maintenance three-dimension contraction screen assays with CAF transfected by the 50 RNAi and subsequently transferred in collagen gel (n=2 in triplicate). **E.** Raw data from all three collagen contraction assays screening.

See Supplementary File 1

Supplementary Table S2: 50 selected genes list siRNA oligonucleotides list

List of smart pool RNA oligonucleotides for each of the 50 selected genes.

See Supplementary File 2
